# Relationship between the neutrophil to high-density lipoprotein cholesterol ratio and severity of coronary artery disease in patients with stable coronary artery disease

**DOI:** 10.3389/fcvm.2022.1015398

**Published:** 2022-11-24

**Authors:** Jie Gao, Jun Lu, Wenjun Sha, Bilin Xu, Cuiping Zhang, Hongping Wang, Juan Xia, Hong Zhang, Wenjun Tang, Tao Lei

**Affiliations:** ^1^Department of Endocrinology, Putuo Hospital, Shanghai University of Traditional Chinese Medicine, Shanghai, China; ^2^Department of Cardiology, Putuo Hospital, Shanghai University of Traditional Chinese Medicine, Shanghai, China; ^3^Heart Function Examination Room, Tongji Hospital, Tongji University, Shanghai, China

**Keywords:** neutrophil, inflammation, low-density lipoprotein cholesterol (LDL-C), high-density lipoprotein cholesterol (HDL-C), coronary artery disease

## Abstract

**Objective:**

To evaluate the link between the neutrophil to HDL-C ratio (NHR) and the degree of coronary stenosis in patients with stable coronary artery disease (CAD).

**Materials and methods:**

Totally 766 individuals who attended our clinic for coronary angiography between January 2019 and January 2021 were included in this study. The participants were divided into two groups, including the CAD group and control group. Spearman correlation analysis was used to investigate the association between NHR and Gensini score and logistic regression analysis was performed to determine the influence of NHR on CAD and severe CAD. Receiver operating characteristic (ROC) curve was constructed to analyze the predictive value of NHR for severe CAD.

**Results:**

The CAD group had a substantially higher median NHR than the control group (3.7 vs. 3.2, *P* < 0.01). There was a positive correlation between NHR and Gensini score, as well as the frequency of coronary artery plaques. Logistic regression demonstrated that NHR was an independent contributor for CAD and severe CAD. In ROC analysis, the area under the ROC curve (AUC) for NHR was larger than that for neutrophil, HDL-C or LDL-C/HDL-C, and the differences were statistically significant (all *P* < 0.05). The NHR limit that offered the most accurate prediction of severe CAD according to the greatest possible value of the Youden index, was 3.88, with a sensitivity of 62.6% and a specificity of 66.2%.

**Conclusion:**

NHR was not only associated with the occurrence and seriousness of CAD, but also a better predictor of severe CAD than neutrophil, HDL-C or LDL-C/HDL-C.

## Introduction

Coronary artery disease (CAD) is a global epidemic disorder with high morbidity and mortality (1). With increasing urbanization, changes in lifestyle and population aging, the incidence of CAD is expected to increase globally in the next decade. The development of atherosclerosis is a primary contributor to the pathophysiology of CAD, and studies have shown that inflammation and lipid metabolic disorders are closely related to atherosclerosis (1–6).

Traditional inflammatory parameters such as White blood cell (WBC) and its subtypes, including neutrophil to lymphocyte ratio (NLR), monocyte to lymphocyte ratio (MLR), platelet to lymphocyte ratio (PLR), monocyte to high density cholesterol ratio (MHR) and mean platelet volume to lymphocyte ratio (MPVLR) (7–13) are commonly used to assess inflammation and calculate the potential for cardiovascular events. Additionally, these parameters are effective indicators of CAD initiation and advancement (7, 9, 10, 14). There is also a significant negative relationship between levels of HDL-C and the risk of CAD, with a better prognosis for CAD related to having a high HDL-C level (15). It has been demonstrated that HDL-C is advantageous in a variety of ways, including its ability to reduce the risk of cardiovascular disease, for example, stimulating cholesterol efflux and reversing cholesterol transport (16, 17), decreasing endothelial activation (18), preventing LDL-C oxidation (19), etc. Furthermore, previous studies indicated that activated neutrophils could not only have an impact on the composition and function of HDL-C (20), but its function could also be regulated by HDL-C (21).

As mentioned above, atherogenesis is facilitated by several factors, including neutrophils and HDL-C, therefore, it was hypothesized that the neutrophil to HDL-C ratio (NHR) is an integrated biomarker more relevant to atherosclerosis as it would offer information about both inflammation and lipid metabolism. For this reason, our study investigated the connection between NHR and CAD and develop the projected value of NHR for the grading of coronary stenosis in individuals with stable CAD.

## Materials and methods

### Study design and subjects

**FIGURE 1:**
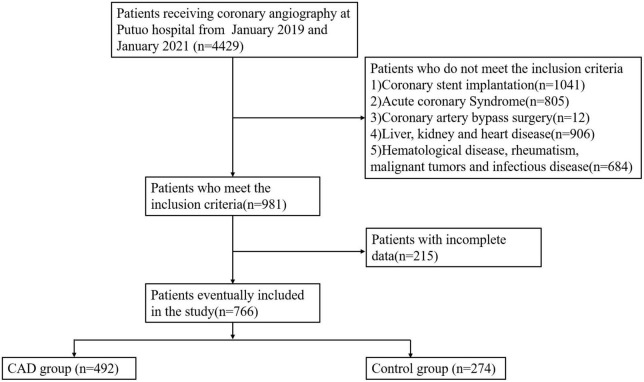
Flow chart of the research process.

This retrospective study was conducted in a single center at Putuo hospital, which is affiliated to the Shanghai University of Traditional Chinese Medicine. 4,429 participants presented with CAD-related symptoms such as chest tightness or pain between January 2019 and January 2021 underwent coronary angiography. Individuals with acute coronary syndrome, coronary stent implantation, liver and kidney disease, infectious disease, severe heart failure, valvular heart disease, hematological diseases, rheumatic diseases, malignant tumors, who had undergone bypass surgery of their coronary arteries and for whom there were no medical records when the study was conducted were excluded. Finally, 766 patients participated in this study. CAD patients had one of the main coronary arteries or more with greater than 50% lumen stenosis (*n* = 492), whereas controls had no luminal stenosis or lower than 30% (*n* = 274) (Figure 1).

The study was approved by the institutional review board of Shanghai University of Traditional Chinese Medicine’s Putuo hospital and was conducted according to the principles articulated in the second version of the Helsinki Declaration. All participants provided written informed consent and their individual information was kept strictly confidential.

### Anthropometric and biochemical measurements

At the time of admission, the patient’s anthropometric measurements and biochemical variables were recorded. After subjects had rested for 10 min and been seated, their blood pressure was measured twice. The mean blood pressure was then calculated. All patients’ height and weight were measured to calculated the body mass index (BMI) by the formula of weight/height2 (kg/m^2^). All patients were required to fast the previous night before having peripheral venous blood samples taken for measurement of CRP, FBG, PBG, TC, TG, HDL-C, LDL-C, WBC, neutrophils, and serum creatinine automatically using a Beckman Colter AU5800 biochemical analyzer (Brea, CA, USA). The CKD-EPI formula was used to derive estimated Glomerular Filtration Rate (eGFR) (22). HbA1c was measured by high-performance liquid chromatography on a Tosoh Automated Glycohemoglobin Analyzer HLC-723G11(Shunan, Yamaguchi, Japan). LDL-C/HDL-C was calculated by taking LDL-C (mmol/l) and dividing it by HDL-C (mmol/l), while NHR was determined by dividing the neutrophil counts (in 10^9^/l) by HDL-C (in mmol/l).

Systolic blood pressure (SBP) ≥ 140 mmHg and/or diastolic blood pressure (DBP) ≥ 90 mmHg (23) were used as the criteria for the diagnosis of hypertension and/or the need for the treatment with antihypertensive drugs. Type 2 diabetes mellitus(T2DM) was defined by the American Diabetes Association in 2021 (24) as follows: FBG ≥ 7.0 mM, 2-h PBG ≥ 11.1 mM and/or HbA_1c_ ≥ 6.5% or the subjects who are using glucose-controlling medicines.

### Coronary angiography and assessment of severity of coronary artery disease

All participants underwent coronary angiography with the cardiologist blinded to the patient’s details. At least 50% stenosis in one of the four main coronary arteries (left main, anterior descending, left circumflex, and right coronary) was required to diagnose CAD. The coronary artery stenosis disorders were classified as single-vessel (*n* = 178), double-vessel (*n* = 154), and triple-vessel (*n* = 160) forms based on the number of affected coronary arteries. The Gensini score was utilized to determine the degree of CAD intensity, which was calculated by summing the values for the location and luminal narrowing of each lesion (Table 1). The participants were divided into three categories (tertiles) based on their Gensini scores: tertile 1 ≤ 24 points (*n* = 180), tertile 2 from 24–45 points (*n* = 149), and tertile 3 had a score ≥ 45 points (*n* = 163). Patients with a Gensini score ≥ 45 points were considered to have severe CAD.

**TABLE 1:** The Gensini score system.

The degree of stenosis (%)	Score	Lesion location	Score
1–25	1	The left main coronary artery	5.0
26–50	2	The proximal left anterior descending artery or proximal left circumflex artery	2.5
51–75	4	The mid-region of the left anterior descending artery	1.5
76–90	8	The distal left anterior descending artery, first diagonal branch	1.0
91–99	16	The mid-distal region of the left circumflex artery	1.0
100	32	Right coronary artery	1.0
		The second diagonal and other segments	0.5

### Statistical analysis

All statistical analyses were performed using SPSS version 22.0. The Kolmogorov–Smirnov test was used to validate the distribution of continuous variables. The mean and SD of regularly distributed variables are presented, whereas the median is provided for non-normally distributed data (interquartile range). Categorical variables are represented numerically and statistically in numbers and percentages. The independent samples *t*-test and the Mann–Whitney U test were used to assess the data, depending on whether or not they followed a normal distribution. For comparison among three groups, ANOVA or Kruskal–Wallis (KW) test was performed according to the nature of the variables. Chi–square test was used to analyse the statistical significance of differences between groups of categorical variables. The relationship between NHR and CAD was investigated using multivariable logistic regression and a ROC curve was used to evaluate the sensitivity and specificity of NHR’s ability to predict severe CAD. The maximum Youden index was utilized to establish the optimal cut-off for the NHR. MedCalc was used to compare the AUCs for NHR, neutrophil, HDL-C and LDL-C/HDL-C. The level of statistical significance was determined to be a two-tailed *P* < 0.05.

## Results

### Baseline patient characteristics

Table 2 displays the demographic and biochemical variables for the 766 patients. Compared to the control group, the prevalence of males, hypertension, diabetes, smoking, and the use of medication was considerably greater among those with CAD (all *P* < 0.01). Besides, subjects with CAD were of older age and had higher SBP, serum creatinine and poorer glycemic management than their counterparts (all *P* < 0.01). When comparing CAD patients with non-CAD patients, the total WBCs and neutrophil counts were higher, whereas levels of TC, TG, HDL-C, and LDL-C were lower in the former group. The CAD group also had higher median NHR levels (3.7 vs. 3.2, *P* < 0.01). BMI, DBP, CRP, LDL-C/HDL-C, and frequency of alcohol consumption were not different between the two groups.

**TABLE 2:** Baseline characteristics of the study population.

	All (*n* = 766)	Control (*n* = 274)	CAD (*n* = 492)	*P*-value
Age (years)	61.4 ± 9.8	58.3 ± 9.7	63.2 ± 9.4	0.000
Male (%)	494 (64.5%)	131 (47.8%)	363 (73.8%)	0.000
BMI (kg/m^2^)	25.0 ± 3.3	25.0 ± 3.6	25.1 ± 3.2	0.470
Smoking (%)	213 (27.8%)	57 (20.8%)	156 (31.7%)	0.001
Drinking (%)	92 (12.0%)	32 (11.7%)	60 (12.2%)	0.908
Diabetes (%)	181 (23.6%)	34 (12.4%)	147 (29.9%)	0.000
Hypertension (%)	473 (61.7%)	155 (56.6%)	318 (64.6%)	0.030
SBP (mmHg)	136.1 ± 19.8	132.0 ± 17.9	138.3 ± 20.5	0.000
DBP (mmHg)	76.7 ± 11.4	77.6 ± 11.2	76.3 ± 11.5	0.186
WBC (10^9^/L)	6.2 (5.3–7.3)	6.0 (5.1–7.1)	6.4 (5.3–7.4)	0.001
Neutrophils (10^9^/L)	3.6 (2.9–4.5)	3.4 (2.7–4.2)	3.7 (3.1–4.7)	0.000
CRP (mg/L)	0.8 (0.4–2.1)	0.8 (0.4–1.9)	0.9 (0.4–2.7)	0.065
FBG (mmol/L)	5.1 (4.6–6.0)	4.9 (4.6–5.4)	5.2 (4.6–6.3)	0.000
PBG (mmol/L)	7.5 (6.0–10.5)	6.8 (5.6–8.4)	8.2 (6.3–11.9)	0.000
HbA1c (%)	5.9 (5.6–6.6)	5.8 (5.5–6.1)	6.0 (5.7–7.1)	0.000
TC (mmol/L)	4.1 (3.5–4.9)	4.3 (3.6–5.1)	4.0 (3.4–4.8)	0.001
TG (mmol/L)	1.5 (1.1–2.0)	1.5 (1.1–2.0)	1.4 (1.1–2.0)	0.868
HDL-C (mmol/L)	1.0 (0.9–1.2)	1.1 (0.9–1.3)	1.0 (0.9–1.2)	0.000
LDL-C (mmol/L)	2.4 (1.8–3.0)	2.6 (1.9–3.1)	2.3 (1.8–3.0)	0.028
Creatinine (umol/L)	78.0 (68.0–87.0)	73.0 (64.0–83.0)	80.0 (70.0–90.0)	0.000
eGFR	87.9(76.7–96.1)	90.5(81.2–98.3)	86.1(74.4–94.8)	0.000
NHR	3.5 (2.6–4.6)	3.2 (2.3–4.2)	3.7 (2.8–4.9)	0.000
LDL-C/HDL-C	2.3 (1.7–3.1)	2.2 (1.7–3.0)	2.4 (1.8–3.1)	0.102
Medications				
Aspirin (%)	650 (84.9%)	206 (75.2%)	444 (90.2%)	0.000
Statins (%)	629 (82.1%)	166 (60.6%)	463 (94.1%)	0.000
Hypoglycemic drugs (%)	140 (18.3%)	29 (10.6%)	111 (22.6%)	0.000

Data were expressed as mean ± standard deviation for normally distributed continuous variables, median (interquartile range) for abnormally distributed variables and number (%) for category variables. For comparisons between groups, the independent samples *t*-test was applied for normally distributed continuous variables and Mann–Whitney U test was used for skewed data. For categorical data, χ^2^ test or Fisher’s exact test were used to test the differences between groups.

BMI, body mass index; SBP, systolic blood pressure; DBP, diastolic blood pressure; WBC, white blood cell; CRP, C reactive protein; TC, total cholesterol; TG, triglyceride; HDL-C, high-density lipoprotein cholesterol; LDL-C, low-density lipoprotein cholesterol; eGFR, estimated Glomerular Filtration Rate; FBG, fasting blood glucose; PBG, postprandial blood glucose; HbA1c, hemoglobin A1c.

Statin includes rosuvastatin, simvastatin, pravastatin, etc. Hypoglycemic drugs include metformin, sulfonylureas, glucosidase inhibitors, glinides.

Table 3 summarized the baseline characteristics of the population stratified by tertiles of Gensini score. *P* for trend was calculated with each tertile of Gensini score taken as a unit. The participants with the highest tertile of Gensini score had higher levels of BMI, WBCs, neutrophils, CRP, HbA1c, Cr, NHR, and higher prevalence of diabetes, but lower levels of HDL-C and less alcohol drinkers, compared to those with the lowest tertile of Gensini score (all *P* for trend < 0.05).

**TABLE 3:** Baseline characteristics of the participants stratified by tertiles of Gensini score.

	Gensini score			
	Tertile 1 (≤ 24)	Tertile 2 (24–45)	Tertile 3 (≥ 45)	*P*-trend
*n* (%)	180 (36.6%)	149 (30.3%)	163 (33.1%)	/
Age (years)	62.1 ± 8.9	64.1 ± 9.9	63.5 ± 9.5	0.146
Male (%)	129 (71.7%)	107 (71.8%)	127 (77.9%)	0.341
BMI (kg/m^2^)	24.7 ± 3.3	24.7 ± 2.9	25.8 ± 3.3*^#^	0.001
Smoking (%)	64 (35.6%)	39 (26.2%)	53 (32.5%)	0.184
Drinking (%)	35 (19.4%)	15 (10.1%)	10 (6.1%)*	0.001
Diabetes (%)	42 (23.3%)	42 (28.2%)	63 (38.7%)*	0.007
Hypertension (%)	115 (63.9%)	98 (65.8%)	105 (64.4%)	0.936
SBP (mmHg)	137.3 ± 21.9	138.9 ± 18.5	138.9 ± 20.7	0.695
DBP (mmHg)	75.7 ± 11.3	76.2 ± 11.5	76.9 ± 11.7	0.650
WBC (10^9/L)	6.1 (5.1–7.2)	6.2 (5.1–7.2)	6.9 (5.7–8.1)*^#^	0.000
Neutrophils (10^9/L)	3.5 (2.9–4.3)	3.5 (3.0–4.6)	4.1 (3.4–5.2)*^#^	0.000
CRP (mg/L)	0.6 (0.4–1.6)	0.9 (0.5–2.0)	1.5 (0.6–4.3)*	0.000
FBG (mmol/L)	5.1 (4.6–6.0)	5.2 (4.7–6.4)	5.3 (4.7–6.4)	0.738
PBG (mmol/L)	7.5 (5.9–10.6)	8.4 (6.4–12.9)	8.7 (6.5–11.7)	0.050
HbA1c (%)	5.9 (5.6–6.6)	6.1 (5.7–6.9)	6.2 (5.7–7.6)*	0.019
TC (mmol/L)	4.0 (3.3–4.7)	4.1 (3.5–4.8)	4.0 (3.4–4.7)	0.624
TG (mmol/L)	1.4 (1.1–2.1)	1.4 (1.0–1.9)	1.5 (1.1–2.0)	0.819
HDL-C (mmol/L)	1.0 (0.9–1.2)	1.1 (0.9–1.2)	0.9 (0.8–1.1)*^#^	0.000
LDL-C (mmol/L)	2.3 (1.7–2.9)	2.4 (1.8–3.1)	2.4 (1.8–3.0)	0.830
Creatinine (umol/L)	78.0 (68.0–86.8)	79.0 (71.0–89.0)	82.0 (71.0–93.0)*	0.042
eGFR	89.1 (79.0–95.6)	86.1 (72.1–93.8)	83.1 (71.7–95.2)	0.065
NHR	3.4 (2.7–4.2)	3.7 (2.6–5.0)	4.2 (3.3–6.0)*^#^	0.000
LDL-C/HDL-C	2.2 (1.7–2.9)	2.4 (1.7–3.1)	2.5 (1.9–3.2)	0.090
Medications				
Aspirin (%)	157 (87.2%)	138 (92.6%)	149 (91.4%)	0.215
Statins (%)	168 (93.3%)	144 (96.6%)	151 (92.6%)	0.278
Hypoglycemic drugs (%)	37 (20.6%)	32 (21.5%)	42 (25.8%)	0.479

Data were expressed as mean ± standard deviation for normally distributed continuous variables, median (interquartile range) for abnormally distributed variables and number (%) for category variables. ANOVA or Kruskal–Wallis (KW) test was performed among groups for continuous variables and Chi–square test was used for categorical variables.

BMI, body mass index; SBP, systolic blood pressure; DBP, diastolic blood pressure; WBC, white blood cell; CRP, C reactive protein; TC, total cholesterol; TG, triglyceride; HDL-C, high-density lipoprotein cholesterol; LDL-C, low-density lipoprotein cholesterol; eGFR, estimated Glomerular Filtration Rate; FBG, fasting blood glucose; PBG, postprandial blood glucose; HbA1c, hemoglobin A1c.

Statin includes rosuvastatin, simvastatin, pravastatin, etc. Hypoglycemic drugs include metformin, sulfonylureas, glucosidase inhibitors, glinides. *p < 0.05 vs. tertile 1; #*p* < 0.05 vs. tertile 2.

### NHR is associated with CAD and severe CAD

It was established that NHR is a separate risk factor for CAD and severe CAD. Table 4 shows that in the unadjusted model, total NHR was positively correlated to CAD (OR = 1.37, 95% CI 1.23–1.52) and severe CAD (OR = 1.42, 95% CI 1.27–1.59). These significant links were maintained (all *P* < 0.01) after controlling for characteristics such as gender, age, BMI, smoking status, diabetes and hypertension (Model 2), or after accounting for LDL-C, creatinine, HbA1c, and CRP levels (Model 3), and after accounting for medications (Model 4). In the unadjusted model, each 1SD increase in NHR level was significantly associated with 1.73-folds (95% CI 1.44–2.08) increased risk of CAD and 1.85-folds (95% CI 1.52–2.25) increased risk of severe CAD. The adjusted ORs (95% CI) for CAD and severe CAD were 1.55 (1.27–1.91) and 1.84 (1.50–2.26) in Model 2, 1.50 (1.21–1.86) and 1.86 (1.50–2.30) in Model 3, 1.41 (1.12–1.77) and 1.85 (1.50–2.29) in Model 4 (all *P* < 0.01).

**TABLE 4:** Association of NHR levels with coronary artery disease (CAD) and severe CAD.

NHR	CAD				Severe CAD			
	Total		SD		Total		SD	
	OR	95% CI	OR	95% CI	OR	95% CI	OR	95% CI
Model 1^a^	1.37	1.23–1.52	1.73	1.44–2.08	1.42	1.27–1.59	1.85	1.52–2.25
Model 2^b^	1.29	1.14–1.44	1.55	1.27–1.91	1.42	1.26–1.59	1.84	1.50–2.26
Model 3^c^	1.26	1.12–1.42	1.50	1.21–1.86	1.42	1.26–1.61	1.86	1.50–2.30
Model 4^d^	1.21	1.07–1.38	1.41	1.12–1.77	1.42	1.26–1.60	1.85	1.50–2.29

Data were obtained using a binary logistic regression model.

^a^Unadjusted.

^b^Adjusted for sex, age, BMI, hypertension, diabetes, and smoking status.

^c^Model 2 and additional adjustment for LDL-C, HbA1c, Cr, and CRP.

^d^Model 3 and additional adjustment for intake of aspirin, statin and hypoglycemic drugs.

NHR, neutrophil to HDL-C ratio; CI, confidence interval; SD, standard deviation.

### Relationship between NHR and the severity of coronary artery disease

**FIGURE 2:**
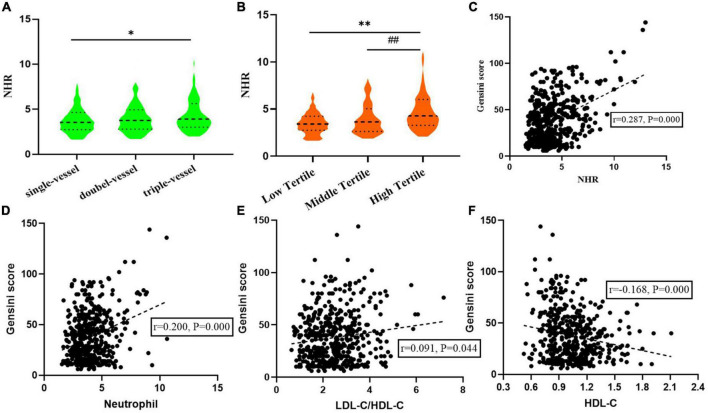
Relationship between neutrophil to HDL-C ratio (NHR) and the severity of coronary artery disease. **(A)** Relationship between NHR and the number of coronary artery lesions. **(B)** NHR levels from low to high tertile groups according to Gensini score. **(C–F)** Correction between NHR, Neutrophil, LDL-C/HDL-C, HDL-C, and Gensini score. *P < 0.01 compared with single-vessel group. ^**^P < 0.01 compared with low tertile group. ##*P* < 0.01 compared with middle tertile group.

As shown in Figure 2A, an incremental increase in NHR was found with the increasing number of coronary artery lesions. Median NHR levels in the single, double, and multiple vessel disease groups were 3.55 (2.74–4.64), 3.76 (2.79–4.94) and 3.91 (3.00–5.63), respectively, indicating significant differences (*P* < 0.05). According to the tertiles of Gensini score, median NHR levels from low tertile to high tertile groups were 3.42 (2.75–4.25), 3.64 (2.64–5.05), and 4.28 (3.28–6.03), respectively. The high tertile group also had NHR levels that were noticeably higher than the middle tertile group as well as the low tertile group (Figure 2B). In addition, Spearman correlation analysis revealed that NHR exhibited moderate correlation with Gensini scores (r = 0.287, *P* = 0.000). The scatter diagram was shown in Figure 2C. And a weak correlation was found between neutrophil (r = 0.200, *P* = 0.000), LDL-C/HDL-C (r = 0.091, *P* = 0.044) and HDL-C (r = –0.168, *P* = 0.000), with the Gensini score (Figures 2D–F).

### Predictive value of neutrophil to HDL-C ratio (NHR) for the severity of coronary artery disease

**FIGURE 3:**
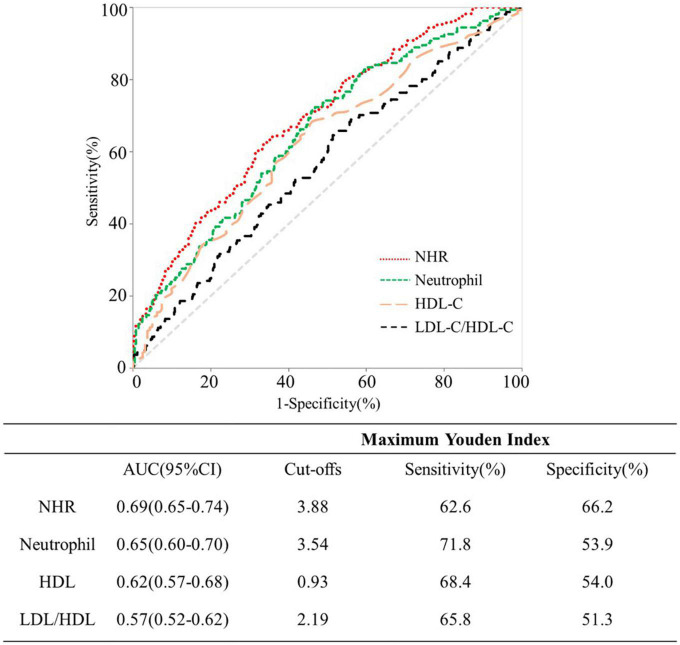
Receiver operating characteristic curves for predicting severe coronary artery disease (CAD).

Figure 3 shows the ROC curves of NHR, neutrophil, HDL-C and LDL-C/HDL-C for predicting severe CAD. Comparing NHR with neutrophil, HDL-C and LDL-C/HDL-C based ROC curves, the AUC for NHR was greater, and the differences was statistically significant (all *P* < 0.05). Analysis of ROC curve indicated that 3.88 was the optimal threshold of NHR in detecting severe CAD, which accorded with 62.6% sensitivity and 66.2% specificity.

## Discussion

According to the reports, CAD accounts for 44.60% of all deaths in rural areas and 42.51% in urban areas, respectively (25), leading to the major cause of mortality worldwide (26). Plasma inflammatory biomarkers aid in predicting a future CAD event and are involved in the onset, development, and instability of atherosclerotic plaques (27–29). Recent studies also focused more on the association between lipid-related biomarkers and CAD (30). Our study suggested that the integrated biomarker NHR (the combination of inflammation and lipid metabolism level) may be more comprehensive and practical in predicting the initiation, development, and prognosis of CAD. Adjusting for potential confounders, NHR was a significant risk factor for CAD and severe CAD. Furthermore, the predicative value of NHR was superior to neutrophil, HDL-C or LDL-C/HDL-C for severe CAD, with a plasma NHR > 3.88 predicting severe CAD with a sensitivity of 62.6% and a specificity of 66.2%.

There was a correlation between high NHR and severe CAD, possibly because inflammation is involved in the pathogenesis of atherosclerosis, which is largely mediated by circulating neutrophils (31). The role of neutrophils in the process of atherosclerosis has been reported (32) and they were considered as a marker of inflammation as well as a predictor of cardiovascular risk (33). Neutrophils were abundant in coronary artery lesions (34) and increased neutrophil counts were associated with an elevated risk of cardiac complications (35). Furthermore, a previous study confirmed that neutrophil count was associated with the complex coronary stenosis and was an independent predictor of multiple complex stenosis (33). In addition, we found that the neutrophil level in the CAD group was significantly higher than that in the control group and the neutrophil correlated positively with Gensini score. However, our research did not find the statistical difference in CRP levels between the two groups. This could reasonably be associated with the use of statins. Studies have reported that statins could suppress the production of CRP in the liver by interrupting the production of IL-6; decrease the CRP levels produced in the atherosclerotic plaques; reduce the CRP-stimulating factor such as E-selectin, intercellular adhesion molecule-1 (ICAM-1), and vascular cell adhesion molecule-1 (VCAM-1) on the activated endothelial cells (36, 37).

A negative correlation between both myocardial infarction (MI) and CAD with HDL-C was observed (38). A moderate increase in HDL-C may even reduce cardiovascular risk to a certain extent (39–41). HDL-C exerted its atheroprotective effect in multiple ways, such as reverse cholesterol transport (RCT) activity, anti-oxidant and anti-inflammatory properties, endothelial protection, etc., (42, 43). Crucially, HDL-C may be related to the lipid raft abundance by inhibiting neutrophils activation, attachment, diffusion, and migration (31). In our study, we also found that HDL-C was negatively associated with Gensini score, but the lipid levels in CAD group were lower than that in control group. A possible explanation for this is that people with CAD are more likely to take statins than those without CAD (94.1% vs. 60.6%, *P* < 0.01).

According to the above analysis, neutrophil increases and HDL-C level decreases in CAD patients, suggesting that the comprehensive indicator NHR may make more contribution for CAD. Chen et al. (44) found that NHR was a predictive marker for metabolic syndrome. Despite previous research linking NHR to developing CAD (30), the participants in that study were all older people who had suffered an acute myocardial infarction (AMI). In keeping with the earlier study of Tuli Kou et al. (45), we discovered that NHR was not only linked to coronary artery stenosis, but also an independent factor for CAD. We also compared NHR’s predictive power to that of the neutrophil count, HDL-C and the LDL-C/HDL-C ratio. Moreover, our study assessed the predicative value of NHR for severe CAD (Gensini score ≥ 45 points) and had a relatively larger sample size.

The Gensini score is one of the most regularly used CAD severity markers, with varied weight coefficients (46, 47). We also identified a positive correlation between NHR and Gensini scores in the present investigation. Identifying CAD patients at high risk may be improved by using both the NHR and the Gensini score.

In summary, we believe that NHR is an effective serological biomarker to predict CAD and assess the degree of coronary stenosis. Nonetheless, our research was not without its caveats. First, because our study was conducted at a single center, the findings cannot be generalized. Second, it was a retrospective and observational study, so the causal relationship between NHR and CAD could not be determined. Finally, we only collected NHR levels at admission and did not investigate the prognostic value of NHR on adverse cardiovascular outcomes. Further multi-center and prospective studies are needed to confirm these findings.

## Conclusion

The high ratio of neutrophils to HDL (NHR) was related to an increased risk of serious CAD. Unlike many other biological parameters, NHR may be inexpensively calculated from a complete blood count on admission, so has the potential as a simple clinical marker for the assessment of CAD.

## Data availability statement

The original contributions presented in this study are included in the article/supplementary material, further inquiries can be directed to the corresponding author/s.

## Ethics statement

The studies involving human participants were reviewed and approved by the Institutional Review Board of Shanghai University of Traditional Chinese Medicine’s Putuo hospital. The patients/participants provided their written informed consent to participate in this study.

## Author contributions

JG wrote the manuscript. JL and WS performed the statistical analysis. CZ, HW, and JX participated in the data collection and checked the data. BX contributed to the discussion. HZ evaluated the severity of coronary artery disease. TL and WT participated in the design of this study and edited the manuscript. All authors have participated in the work and have reviewed and agreed with the content of the article.

## Abbreviations

SBP: systolic blood pressure
DBP: diastolic blood pressure
BMI: body mass index
CAD: coronary artery disease
TC: total cholesterol
TG: triglycerides
HDL-C: high-density lipoprotein cholesterol
LDL-C: low-density lipoprotein cholesterol
CRP: C-reactive protein
FBG: fasting blood glucose
PBG: postprandial blood glucose
HbA1c: glycosylated hemoglobin A1c
eGFR: estimated Glomerular Filtration Rate
ROC: receiver operating characteristic curve
AUC: area under the ROC curve
OR: odds ratio
WBC: White blood cell
SD: Standard deviation
NLR: neutrophil to lymphocyte ratio
MLR: monocyte to lymphocyte ratio
PLR: platelet to lymphocyte ratio
MHR: monocyte to high-density cholesterol ratio
RCT: reverse cholesterol transport
MPVLR: mean platelet volume to lymphocyte ratio
MI: myocardial infarction
ICAM-1: intercellular adhesion molecule-1
VCAM-1: vascular cell adhesion molecule-1.
